# Homozygous G650del nexilin variant causes cardiomyopathy in mice

**DOI:** 10.1172/jci.insight.138780

**Published:** 2020-08-20

**Authors:** Canzhao Liu, Simone Spinozzi, Wei Feng, Ze’e Chen, Lunfeng Zhang, Siting Zhu, Tongbin Wu, Xi Fang, Kunfu Ouyang, Sylvia M. Evans, Ju Chen

**Affiliations:** 1 Department of Medicine, UCSD, La Jolla, California, USA.; 2 Drug Discovery Center, State Key Laboratory of Chemical Oncogenomics, School of Chemical Biology and Biotechnology, Peking University Shenzhen Graduate School, Shenzhen, China.; 3 Department of Pharmacology, Skaggs School of Pharmacy and Pharmaceutical Sciences, UCSD, La Jolla, California, USA.

**Keywords:** Cardiology, Genetics, Cardiovascular disease, Excitation contraction coupling, Genetic diseases

## Abstract

Nexilin (NEXN) was recently identified as a component of the junctional membrane complex required for development and maintenance of cardiac T-tubules. Loss of Nexn in mice leads to a rapidly progressive dilated cardiomyopathy (DCM) and premature death. A 3 bp deletion (1948–1950del) leading to loss of the glycine in position 650 (G650del) is classified as a variant of uncertain significance in humans and may function as an intermediate risk allele. To determine the effect of the G650del variant on cardiac structure and function, we generated a G645del-knockin (G645del is equivalent to human G650del) mouse model. Homozygous G645del mice express about 30% of the Nexn expressed by WT controls and exhibited a progressive DCM characterized by reduced T-tubule formation, with disorganization of the transverse-axial tubular system. On the other hand, heterozygous Nexn global KO mice and genetically engineered mice encoding a truncated Nexn missing the first N-terminal actin-binding domain exhibited normal cardiac function, despite expressing only 50% and 20% of the Nexn, respectively, expressed by WT controls, suggesting that not only quantity but also quality of Nexn is necessary for a proper function. These findings demonstrated that Nexn G645 is crucial for Nexn’s function in tubular system organization and normal cardiac function.

## Abstract 2

A mouse model of a dilated cardiomyopathy associated mutation in Nexilin reveals roles in tubular system organization and normal cardiac function.

## Graphical abstract

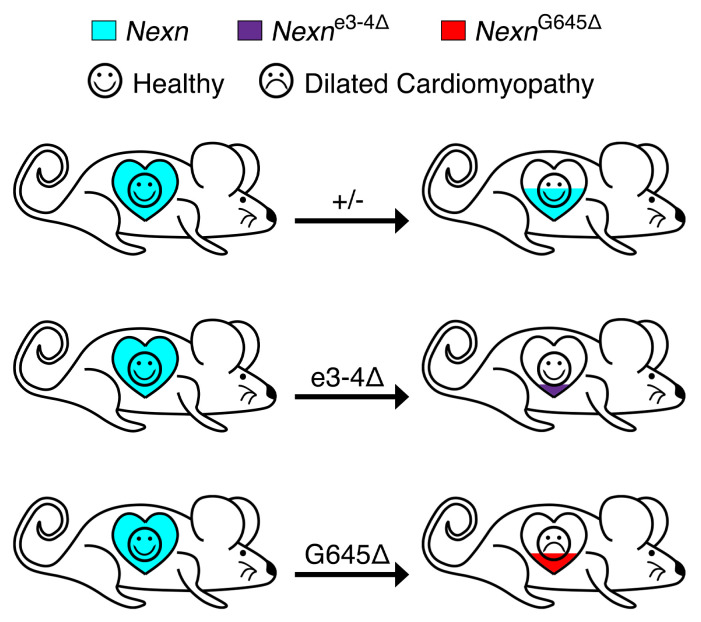

## Introduction

Dilated cardiomyopathy (DCM) is a cardiac disorder defined by enlargement of the cardiac ventricular chambers that leads to systolic dysfunction and heart failure (1–5). Familial DCM is an inherited disease often resulting from genetic transmission of a single gene variant (6–10). The study of monogenic disorders causing heart failure provides an opportunity for defining essential molecules involved in these processes and potentially creates the basis for novel tailored therapies (11–13). Recently, several variants in the nexilin (NEXN) gene have been associated with cardiomyopathy (14, 15). In addition, our previous studies have revealed that cardiomyocyte-specific ablation of *Nexn* results in a rapidly progressive severe DCM, identifying Nexn as a pivotal component of the junctional membrane complex (JMC) required for cardiac T-tubule development and maintenance (16). Nexn interacts with crucial JMC proteins such as Jph2, and the absence of T-tubule formation following Nexn loss in cardiomyocytes is likely due to abnormal expression of calcium-handling proteins and alterations in protein-protein interactions at the JMC (16).

It has been reported that a 3 bp deletion in *NEXN* (1948–1950del), leading to loss of a glycine in position 650 (G650del), was associated with DCM (14). Interestingly, a mutant zebrafish model recapitulated features observed in myocardial biopsies from patients carrying the G650del variant. However, little is known regarding the effect of the G650del variant on cardiac structure and function. To address this issue, we generated a G645del (equivalent to the human G650del) variant–knockin mouse model using CRISPR/Cas9 technology. Comprehensive histological and physiological analyses of this mouse model demonstrated that mice homozygous for the G645del allele expressed only about 30% of the Nexn protein compared with that expressed by WT controls and developed a progressive DCM. More detailed analyses also demonstrated that the G645del variant altered Nexn function in T-tubule formation and organization, ultimately leading to calcium-handling and contractility defects. Interestingly, heterozygous Nexn global KO (gKO) mice and a mutant mouse line carrying a deletion of *Nexn* exon 3 and 4 (e3-4del) had normal heart function, despite expressing 50% and 20% of Nexn, respectively, compared with that expressed by WT controls. Altogether, our results showed that, not only quantity, but also quality, of Nexn is necessary for proper cardiac function and homozygous G645del mice resulted in DCM with transverse-axial tubule system (TATS) defects, demonstrating that the Nexn G645 is crucial for Nexn’s function in mice.

## Results

### Homozygous Nexn G645del variant mice have impaired cardiac function and altered expression of calcium-handling proteins.

**Figure 1:**
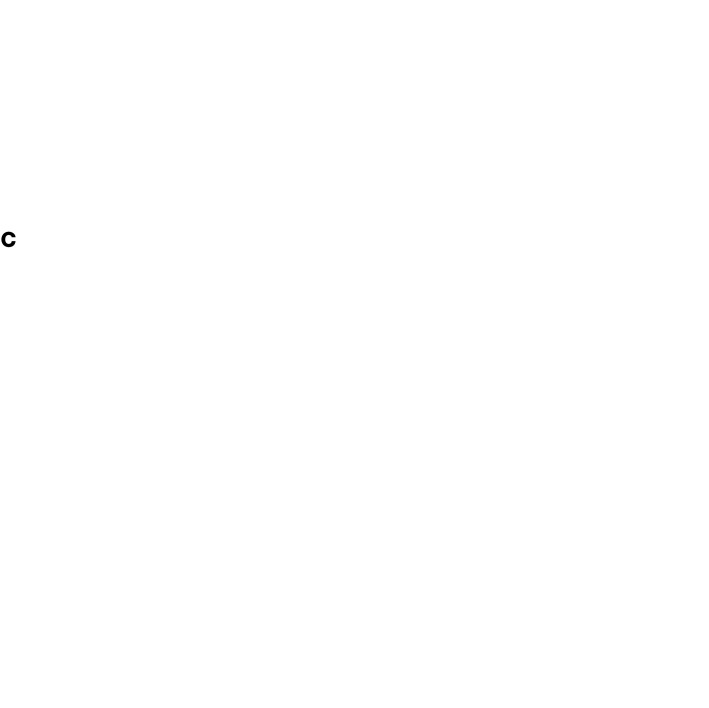
*Nexn*^G645Δ/G645Δ^ mice display progressive dilated cardiomyopathy. (**A**) Schematic representation of Nexn genetic sequence, with glycine in position 645 (G645, equivalent to the human G650). (**B**) Schematic representation of CRISPR/CAS9 deletion strategy. (**C**) Representative PCR from a control (*Nexn*^WT/WT^) mouse and mice homozygous (hom) (*Nexn*^G645Δ/G645Δ^) or heterozygous (het) for the G645 deletion. (**D** and **E**) Representative sequencing of (**D**) a control and (**E**) a *Nexn*^G645Δ/G645Δ^ mouse. (**F**) Representative whole-heart H&E- and Masson’s trichrome–stained images, 4-chamber views, of longitudinal histological sections from *Nexn*^WT/WT^ and *Nexn*^G645Δ/G645Δ^ mice at P10 and 3 months (3M) of age. Scale bars: 1 mm.

**Figure 2:**
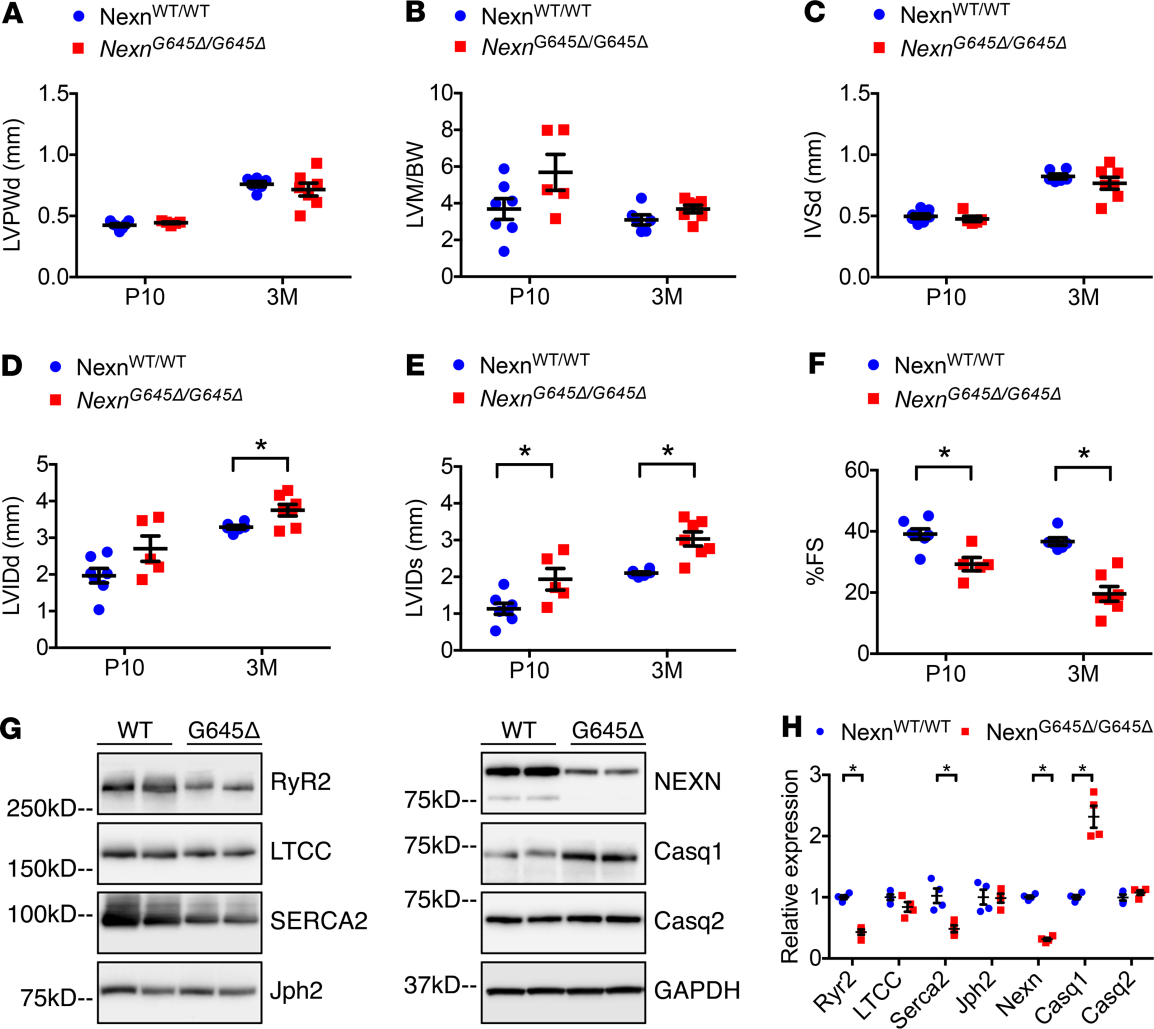
*Nexn*^G645Δ/G645Δ^ mice have impaired cardiac function and calcium-handling protein expression in cardiomyocytes. (**A–F**) Transthoracic echocardiographic measurements from *Nexn*^WT/WT^ and *Nexn*^G645Δ/G645Δ^ mice at P10 and 3 months (3M) of age (*n* ≥ 5): (**A**) left ventricular posterior wall end diastole (LVPWd), (**B**) left ventricular mass-to–body weight ratio (LVM/BW), (**C**) interventricular septal end diastole (IVSd), (**D**) left ventricular internal diameter end diastole (LVIDd), (**E**) left ventricular internal diameter end systole (LVIDs), and (**F**) percentage of fractional shortening (%FS). (**G**) Representative Western blotting images from whole-heart lysates of *Nexn*^WT/WT^ and *Nexn*^G645Δ/G645Δ^ mice at P10, and (**H**) relative quantification normalized to GAPDH. *P < 0.05. Data represent mean ± SEM; statistical significance was analyzed with 2-way ANOVA (**A**–**F**) or 2-tailed Student’s *t* test (**H**).

To investigate the effect of the G650del variant on cardiac structure and function, we created a knockin mouse model in which 3 bp coding for the amino acid residue G645 (equivalent to the human G650) in *Nexn* were deleted from the endogenous gene using CRISPR/Cas9 technology (Figure 1, A–E). The use of the knockin, rather than a transgenic approach, ensured that the mutant *Nexn* gene was under the control of the endogenous locus. Therefore, these knockin mice mimicked the *NEXN* G650del variant found in people. Mice homozygous for the G645del allele (*Nexn*^G645Δ/G645Δ^) exhibited a progressive DCM (Figure 1F). A majority of the *Nexn*^G645Δ/G645Δ^ mice survived until adulthood, with approximately 20% exhibiting periweaning mortality. Indeed, gross anatomical and histological evaluation showed that *Nexn*^G645Δ/G645Δ^ hearts presented with average heart size, with slightly enlarged left ventricle at P10 and a progressive chamber dilation, clearly visible at 3 months of age (Figure 1F). Transthoracic echocardiography analyses of *Nexn*^G645Δ/G645Δ^ and control mice showed no alterations in thickness of left ventricular posterior wall end diastole, calculated ratio of left ventricular mass to body weight, or thickness of interventricular septal end diastole (Figure 2, A–C) but increased left ventricular dimensions in *Nexn*^G645Δ/G645Δ^ hearts and also reduced cardiac function (Figure 2, D–F) at P10 and 3 months of age. Mice with cardiomyocyte-specific KO (cKO) of Nexn die before P12 (16), whereas a majority of *Nexn*^G645Δ/G645Δ^ mice are able to survive to adulthood, albeit with DCM. This suggests that the defective Nexn protein is able to maintain partial function.

Since our previous study demonstrated that loss of Nexn alters expression of some Ca^2+^-handling proteins in the heart (16), we evaluated expression of these Ca^2+^-handling proteins in *Nexn*^G645Δ/G645Δ^ mice. Consistent with our previous data from *Nexn* cKO mice, Western blotting results showed that RyR2, Serca2, and Casq1 had significantly altered expression levels in G645del hearts relative to those of *Nexn*^WT/WT^ controls. Notably, expression of junctophilin 2 (Jph2) and bridging integrator-1 (Bin1) proteins at P10 was not altered in *Nexn*^G645Δ/G645Δ^ hearts (Figure 2, G and H, and Supplemental Figure 1; supplemental material available online with this article; https://doi.org/10.1172/jci.insight.138780DS1).

### Both quantity and quality of Nexn are necessary for normal cardiac function in mice.

Hassel and colleagues showed previously that the G650del variant did not interfere with NEXN mRNA production, assuming also that protein expression was not affected (14). However, this assumption concerning protein expression levels was based on overexpression assays in nonmuscle cells. Consistent with their findings, in our model NEXN mRNA levels showed no significant changes between *Nexn*^WT/WT^ and *Nexn*^G645Δ/G645Δ^ mice (Supplemental Figure 2). On the other hand, protein levels in *Nexn*^G645Δ/G645Δ^ hearts were decreased to 30% of WT levels seen in littermate controls, suggesting that G645del affects Nexn protein stability (Figure 2, G and H).

**Figure 3:**
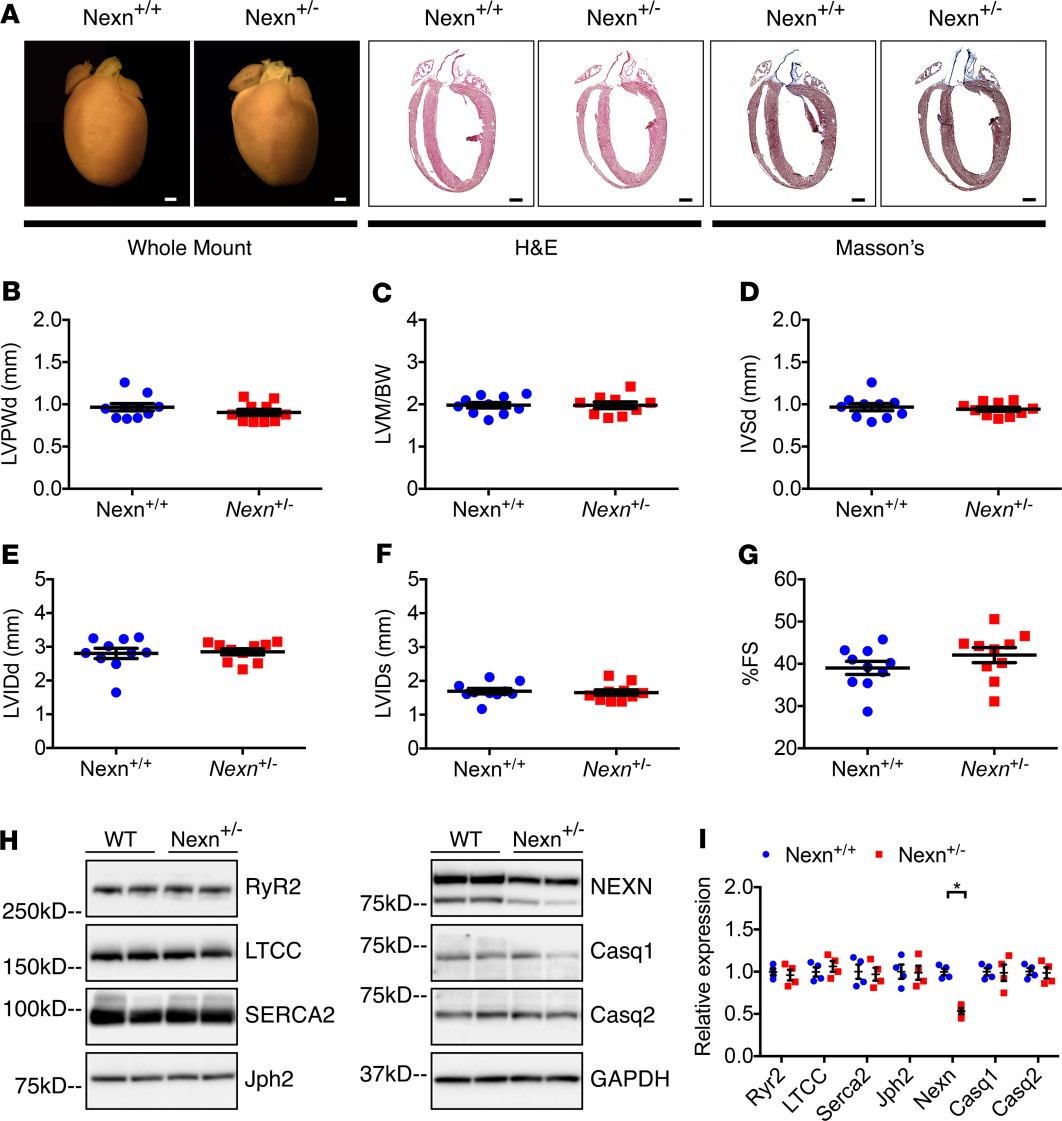
Haploinsufficiency in Nexilin heterozygous gKO mice does not alter cardiac function. (**A**) Representative whole-heart H&E- and Masson’s trichrome–stained images, 4-chamber views, of longitudinal histological sections from *Nexn*^+/+^ and *Nexn*^+/-^ mice at 10 months of age. Scale bars: 1 mm. (**B–G**) Transthoracic echocardiographic measurements from *Nexn*^+/+^ and *Nexn*^+/-^ mice at 10 months of age (*n* ≥ 9): (**B**) Left ventricular posterior wall end diastole (LVPWd), (**C**) left ventricular mass-to–body weight ratio (LVM/BW), (**D**) interventricular septal end diastole (IVSd), (**E**) left ventricular internal diameter end diastole (LVIDd), (**F**) left ventricular internal diameter end systole (LVIDs), and (G) percentage of fractional shortening (%FS). (**H**) Representative Western blotting images from whole-heart lysates of *Nexn*^+/+^ and *Nexn*^+/-^ mice at 10 months of age, and (**I**) relative quantification normalized to GAPDH. *P < 0.05. Data represent mean ± SEM; differences between groups were analyzed by 2-tailed Student’s *t* test.

To evaluate whether the phenotypes observed in *Nexn*^G645Δ/G645Δ^ mice result entirely from 70% downregulation of Nexn G645del protein levels, we conducted a comparison study with heterozygous Nexn gKO mice, which expressed Nexn at 50% of WT levels (Figure 3, H and I). Gross anatomical and histological examination of heterozygous gKO hearts showed no signs of cardiac disease when compared with *Nexn*^+/+^ controls (Figure 3A) at 10 months of age. Echocardiography confirmed that no altered cardiac function or DCM were present (Figure 3, B–G). Ca^2+^-handling proteins, which were significantly changed in *Nexn*^G645Δ/G645Δ^ mice, were not altered in gKO heterozygous mice, indicating that protein levels of 50% of those of WT mice were sufficient to maintain normal heart function, suggesting that a considerable reduction in protein expression was tolerated.

NEXN was first identified as an actin filament-binding protein characterized by 2 actin-binding domains (ABDs) separated by about 130 aa containing a coiled-coil region; hence, NEXN shows F-actin crossing-linking activity (17). It was also reported that the NEXN G650del variant perturbed Z-disc stability, even if no alteration in the NEXN-actin complex was observed (14). Of note, this hypothesis was based on electron microscopy of a >50-year-old patient affected by DCM, and at this stage/age the heart would have already undergone severe remodeling, with sarcomere instability secondary to chronic cardiac stress. To better understand the role of NEXN-actin cross-linking activity, we generated a mouse line carrying e3-4del, where the first N-terminal ABD of Nexn is located, hence disrupting a part of the protein essential for actin cross-linking activity (Supplemental Figure 3, A and B). The amount of truncated Nexn protein found in ventricles of mice homozygous for e3-4del was assessed by Western blot analyses. Although only approximately 20% of truncated Nexn was present with respect to Nexn levels in controls (Supplemental Figure 3C), echocardiographic measurements indicated that left ventricular dimension and systolic function of e3-4del mice was comparable to that of littermate controls (Supplemental Figure 4), indicating that the first N-terminal ABD of Nexn was dispensable for heart function. These results demonstrated that 20% of truncated Nexn protein was sufficient for normal cardiac function and also demonstrated that actin cross-linking activity was not necessary for Nexn cardiac function, as previous studies have demonstrated that both Nexn ABDs are required for actin cross-linking (17) These models together suggest that phenotypes resulting from homozygosity of the G645del protein were at least in part, if not entirely, attributed to altered Nexn protein function consequent to the variant.

### Mouse cardiomyocytes that are homozygous for the Nexn G645del variant have an impaired TATS.

**Figure 4:**
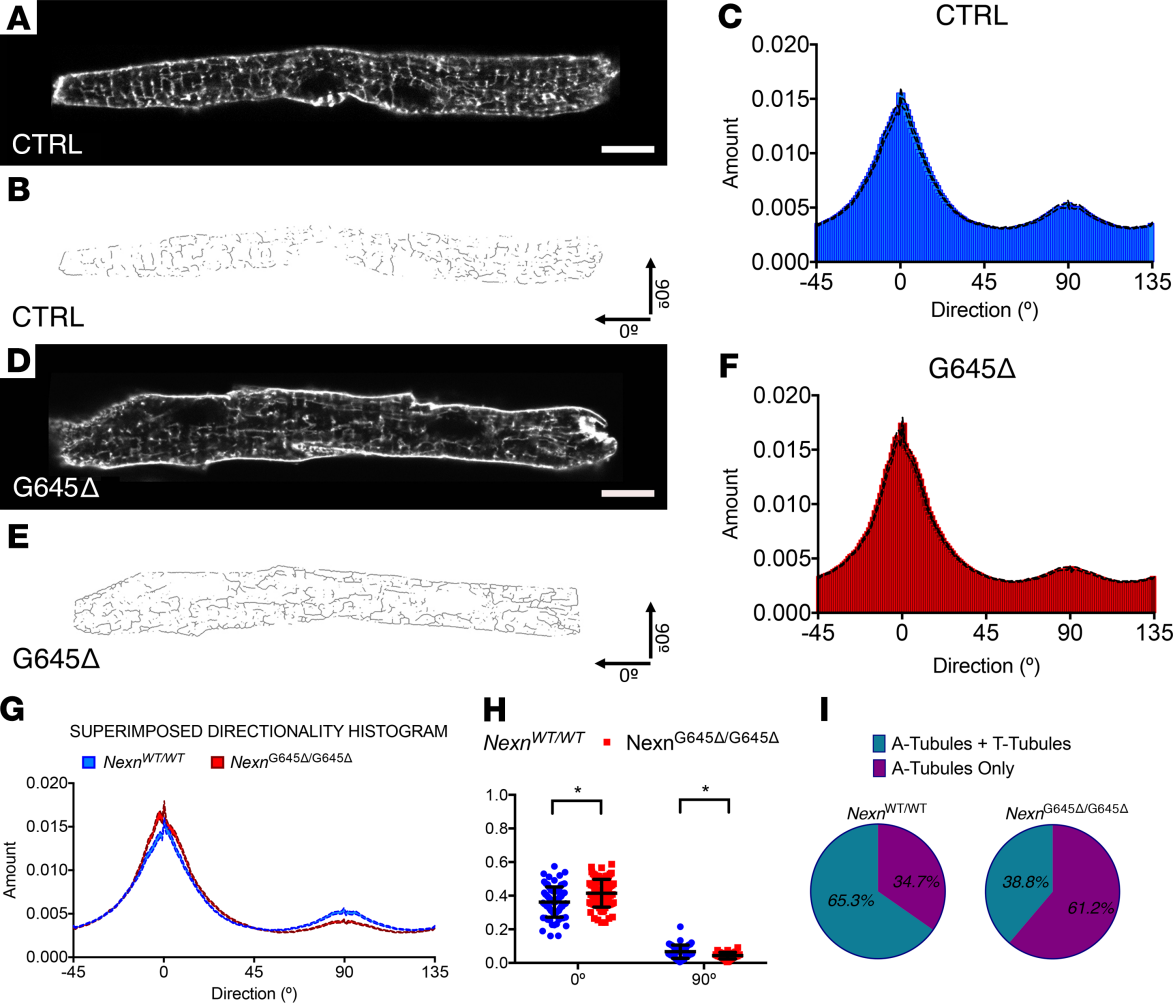
*Nexn*^G645Δ/G645Δ^ mice have a disorganized transverse-axial tubule system. (**A** and **D**) Representative live confocal images of cardiomyocytes stained with Di-8 Anepps isolated from *Nexn*^WT/WT^ and *Nexn*^G645Δ/G645Δ^ mice at P10 (*n* = 3). Scale bar: 10 μm. (**B** and **E**) Relative skeletonized images, oriented in a *x-y* fashion: T-tubules represent the 90° component and axial tubules the 0° component. (**C** and **F**) Directionality histograms derived from Fourier transforms of all the analyzed cardiomyocytes images. (**G**) Comparison between directionality histograms from **C** and **F**. (**H**) *Nexn*^WT/WT^ and *Nexn*^G645Δ/G645Δ^ comparison of area under curve from 90° and 0° tubular Fourier components Gaussian distribution. (**I**) Distribution of cardiomyocytes with a formed transverse-axial tubule system (A-tubules + T-tubules) and immature cardiomyocytes presenting only with a longitudinal tubular component (A-tubules only) in *Nexn*^WT/WT^ and *Nexn*^G645Δ/G645Δ^ mice at P10. *P < 0.05. Data represent mean ± SEM; differences between groups were analyzed by 2-tailed Student’s *t* test.

Given that absence of Nexn in developing cardiomyocytes prevents formation of mature cardiac transverse tubules (T-tubules), ultimately leading to neonatal lethality with severe DCM (16), we decided to further investigate T-tubule organization in *Nexn*^G645Δ/G645Δ^ hearts. In adult murine cardiomyocytes (starting around P10) the invaginated sarcolemma organizes in an intricate network of transverse and longitudinal tubules called TATS. Confocal microscopy live imaging of isolated P10 cardiomyocytes stained with Di-8 Anepps revealed a disorganization of the entire TATS (Figure 4). The T-tubule component (90°) was significantly decreased in *Nexn*^G645Δ/G645Δ^ cardiomyocytes, whereas the longitudinal component (0°) was increased with respect to *Nexn*^WT/WT^ controls, indicating that *Nexn*^G645Δ/G645Δ^ cardiomyocytes had a generally disorganized TATS (Figure 4H). Interestingly, when compared with *Nexn*^WT/WT^ mice, *Nexn*^G645Δ/G645Δ^ mice had a much higher number of cardiomyocytes missing the transverse component of the TATS, suggesting that the *Nexn*^G645Δ/G645Δ^ cardiomyocytes had delayed T-tubule network formation, which usually starts at around P10 in mice.

## Discussion

Variants in NEXN have been associated with cardiomyopathies, highlighting its importance for cardiac function (14, 15). A 3 bp deletion (1948–1950del) leading to loss of glycine at position 650 (G650del) had been reported as being associated with DCM (14), although it subsequently has been classified as a variant of uncertain significance. To investigate the effect of this variant on cardiac structure and function, we created a knockin mouse model in which 3 bp coding for the amino acid residue G645 (equivalent to the human G650) in *Nexn* were deleted. Our data showed that *Nexn*^G645Δ/G645Δ^ mice developed progressive DCM with decreased cardiac function and altered expression of calcium-handling proteins (Figures 1 and 2). Interestingly, levels of Nexn protein in *Nexn*^G645Δ/G645Δ^ hearts were decreased to 30% of WT levels, while mRNA levels were not changed when compared with those of littermate controls, suggesting that G645del affected Nexn protein stability (Figure 2, G and H). However, a 50% decrease in WT Nexn protein, or an 80% decrease of Nexn e3-4del protein, relative to WT Nexn protein levels did not cause any cardiac phenotype (Figure 3 and Supplemental Figures 3 and 4), suggesting that a considerable reduction in Nexn protein expression could be tolerated. These observations suggested that phenotypes resulting from homozygosity of the G645del protein were at least in part, if not entirely, attributed to altered Nexn protein function consequent to the variant.

As *Nexn* gKO mouse cardiomyocytes are impaired in T-tubule formation (16), we analyzed the T-tubular network in *Nexn*^G645Δ/G645Δ^ cardiomyocytes. Our results demonstrated that the TATS was significantly altered and that T-tubule maturation was delayed in *Nexn*^G645Δ/G645Δ^ cardiomyocytes (Figure 4). These findings demonstrated that G645del was required for Nexn’s role in T-tubule formation and TATS organization. Of note, the TATS disorganization observed in *Nexn*^G645Δ/G645Δ^ cardiomyocytes was not related to altered expression of T-tubule proteins, such as Jph2 or Bin1 (Figure 2, G and H, and Supplemental Figure 2) (18–20). Future studies are warranted to understand molecular interactions involved in Nexn-driven TATS organization and to define the precise role of the Nexn C-terminal domain and G645 in this process.

Finally, it should be pointed out that the SNP rs1488731300 (encoding p.Gly650del) is found at 0.02% frequency in the European population (https://gnomad.broadinstitute.org/variant/1-78408430-TGGA-T?data set = gnomad_r2_1) and ClinVar reports Gly650del as a variant of uncertain significance (https://www.ncbi.nlm.nih.gov/clinvar/variation/47899/). Our data that homozygous G645del mice expressed about 30% of Nexn relative to that of WT controls and exhibited a progressive DCM characterized by reduced T-tubule formation with disorganization of the transverse-axial tubular system, demonstrated that NEXN G650 is crucial for NEXN’s function in mice. However, the G650del variant allele in the heterozygous state alone is not sufficient to cause DCM in mice, which is consistent with human population data, as its frequency in the general Europe population is above the credible threshold for pathogenicity. Thus, this variant allele should be considered as a potential intermediate effect risk allele that could modify DCM in the context of other genetic and nongenetic susceptibility factors.

## Methods

### Animal procedures.

Genotyping was performed using primers reported in Supplemental Table 1. *Nexn* G645del mice were generated with CRISPR/CAS9 technology as described previously (21). Briefly, Cas9 proteins (30 ng/μL), crRNA (0.6 pmol/μL), tracrRNA (0.6 pmol/μL), and ssODN (0.6 pmol/μL) were diluted and mixed in IDTE (IDT) buffer. One picoliter of the mixture was then injected into pronuclei of 1-cell-stage zygotes from C57BL/6 J mice (The Jackson Laboratory) after incubation at 37°C for 5 minutes. All mice were backcrossed with C57BL/6J mice for at least 3 generations before experiments.

*Nexn*-floxed mice were generated as previously described (16). Mice heterozygous for the null allele were generated by crossing floxed *Nexn* mice with mice expressing the global Sox2-cre.

The construct used to truncate the first ABD of Nexn was generated by flanking exons 3 and 4 of *Nexn* with 2 LoxP sites. The targeting construct was verified by sequencing and linearized with NotI before electroporation into ES cells at the Transgenic Core Facility of UCSD. G418-resistant ES clones for *Nexn* were screened for homologous recombination by Southern blotting using a previously described method (22). Global expression of the truncated *Nexn* was ensured by crossing the resulting floxed mice with mice expressing global Sox2-cre.

### Echocardiography.

Mice were anesthetized with 3% isoflurane for 10 seconds and maintained at 0.5% isoflurane during the procedure. Echocardiography was performed by using a VisualSonics, SonoSite FUJIFILM, Vevo 2100 ultrasound system with a linear transducer 32–55 MHz.

### Isolation of murine cardiomyocytes.

Cardiomyocytes were isolated from 10-day-old male mice using a Langendorff system as previously reported (16, 23, 24).

### Western blot.

Protein lysates were extracted as previously described (14, 25), separated on 4%–12% SDS-PAGE gels (Life Technologies), and then transferred for 2 hours or overnight at 4°C to a PVDF membrane (Bio-Rad). Membranes were incubated overnight at 4°C with primary antibodies (listed in Supplemental Table 2) after blocking with 5% skimmed milk. Blots were washed and incubated with HRP-conjugated secondary antibody generated in rabbit (1:5000) or mouse (1:5000) (Dako) for 1.5 hours at room temperature. Immunoreactive bands were visualized using an enhanced chemiluminescence reagent (Thermo Fisher Scientific) following manufacturer instructions and then analyzed by densitometry and expressed as pixel density normalized to GAPDH. All buffers used have been previously listed (26).

### Quantitative real-time PCR.

Total RNA and cDNA were extracted as previously described (16). PCR mixture contained 1 μl diluted cDNA, 5 μl 2× iTaq Universal SYBR Green Supermix (Bio-Rad), and 100 nM of each gene-specific primer in a final volume of 10 μl. Real-time PCRs were performed using a CFX96 Bio-Rad Thermocycler. Primers sequences are reported in Supplemental Table 1.

### Whole-heart imaging and histology.

Hearts were excised after euthanasia, perfused with phosphate-buffered saline, and fixed overnight in 4% PFA; they were then processed for whole-heart imaging and histology as previously described (16). For whole-heart imaging, images were acquired with an Olympus SZX12 stereomicroscope. Histology images were acquired with a Hamamatsu Nanozoomer 2.0HT Slide Scanner.

### T-tubule imaging.

Individual cardiomyocytes isolated from 3 different mice of each group were processed and analyzed as previously described (16, 27). Images were processed with Fiji software: skeletonization and directionality analyses were performed using published plugins (28–30). Extrapolated data from Fourier’s components were analyzed using Prism 6.0 (GraphPad Software) to create average directionality histograms and to perform area under curve measurements of the resultant Gaussian curves. Distribution of the mature or immature T-tubule network was also quantified.

### Statistics.

Numeric data are expressed as mean ± SEM. Statistical analysis was performed using Prism 6.0 (GraphPad Software). Differences between groups were analyzed by 2-tailed Student’s *t* test, and a *P* value of less than 0.05 was considered statistically significant.

### Study approval.

The UCSD Animal Care Program maintained all animals, and the UCSD Institutional Animal Care and Use Committee approved animal experimental procedures.

## Author contributions

JC, CL, and SS designed the study. CL, SS, WF, ZC, LZ, SZ, TW, and XF performed the experiments. JC, CL, SS, and SME wrote the manuscript. KO and SME provided advice.

## Supplementary Material

Supplemental data

## Version 1 08/20/2020

Electronic publication
