# Supplementary material for: Homozygous G650del nexilin variant causes cardiomyopathy in mice
Source: JCI Insight. 2020 Aug 20;5(16):e138780. doi: 10.1172/jci.insight.138780 (PMC7455123; doi:10.1172/jci.insight.138780)
Supplement: Supplemental data [file jciinsight-5-138780-s096.pdf]

SUPPLEMENTAL MATERIAL

Homozygous G650del nexilin variant causes cardiomyopathy in mice

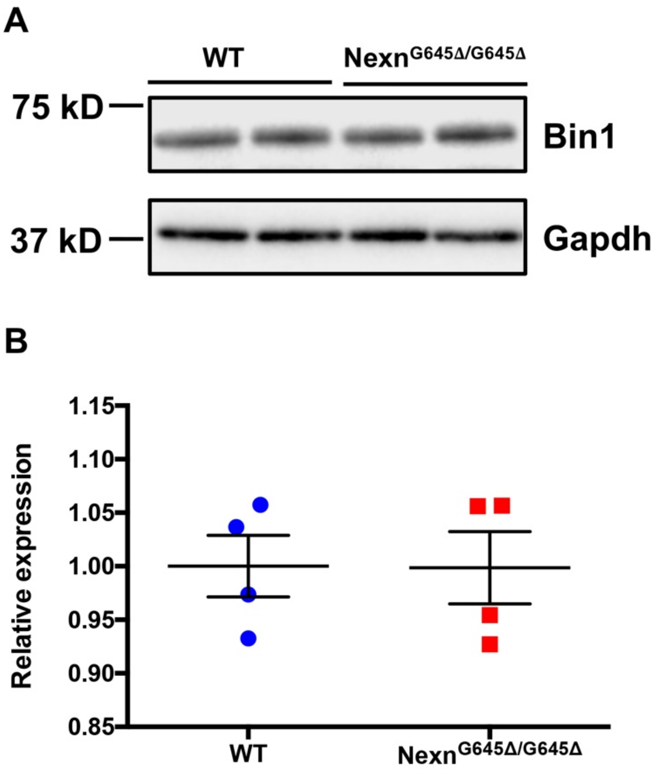

Figure S1. Bridging Integrator-1 protein level is not altered in *Nexn*$^{G645\Delta/\ G645\Delta}$ hearts. (**A**) WB showing no difference in Bridging Integrator-1(Bin1) expression in *Nexn*$^{G645\Delta/\ G645\Delta}$ hearts compared to wild type (WT) control mice and (**B**) relative quantification graph (n= 4, mice age: postnatal day 10).

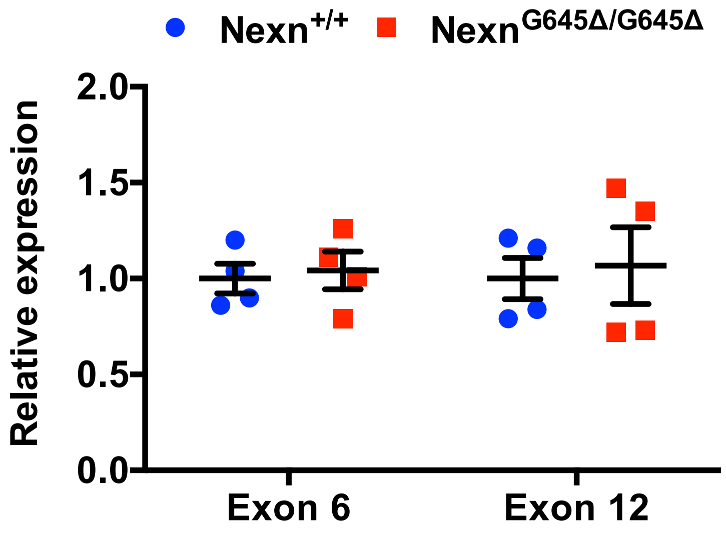

Figure S2. NEXN mRNA expression in *Nexn*$^{+/+}$ and *Nexn*$^{G645\Delta/\ G645\Delta}$ mice. Quantitative PCR using primers for exon 6 and 12 of *Nexn* (n= 4, mice age: postnatal day 10).

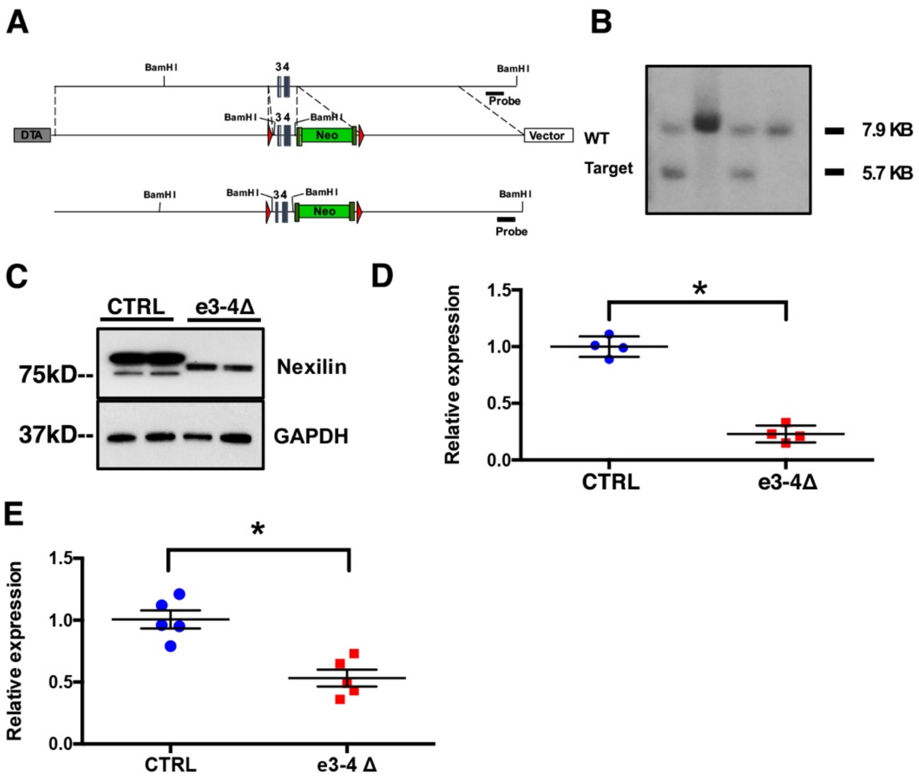

Figure S3. Deletion of exon 3 and 4 of *Nexn* reduce by 80% NEXN expression level. Generation of NEXN exon 3 and 4 deletion floxed mice: (**A**) restriction map of the relevant genomic region of NEXN (top), targeting construct (middle), and the mutated locus following recombination (bottom) (DTA, Diphtheria Toxin A chain gene; Neo, neomycin resistance gene) **(B**) PCR showing successful truncation. (**C**) WB showing decreased NEXN expression in heart homozygous for the exon 3 and 4 deletion (e3-4Δ) compared to wild type control mice (CTRL) and (**D**) relative quantification graph (n=4). (**E**) Quantification graph of mRNA expression levels in Nexn e3-4Δ homozygous mice hearts compared to wild type controls (n=4). Mice age for WB and qPCR analyses is 3 months.

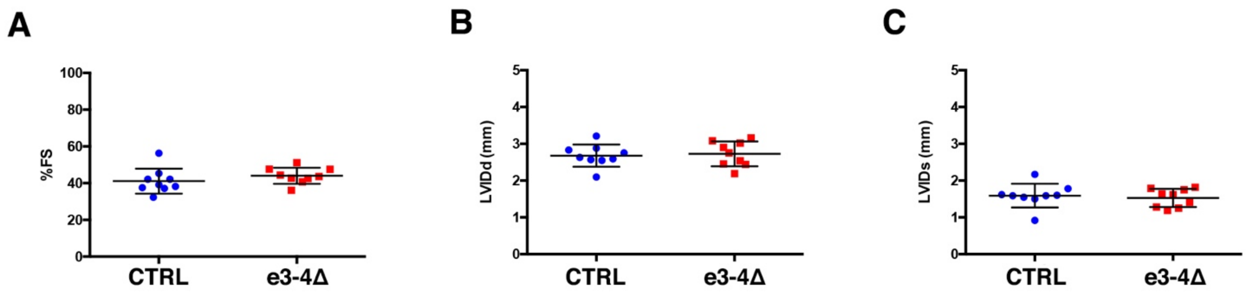

Figure S4. Deletion of exon 3 and 4 of *Nexn* does not alter cardiac function. Echocardiography measurements showing no difference between mice homozygous for the exon 3 and 4 deletion (e3-4Δ) and controls (CTRL) in (**A**) percentage of fractional shortening (%FS), (**B**) left ventricular dimension in end-diastole (LVIDd) and (**C**) left ventricular dimension in end-systole (LVIDs). Mice of 3 months of age n>9.

Primers	5’ -3’	Application
G645 WT-F	5’ -CTTCCCAGAAGATGGAGGA-3’	Genotyping for wild type allele
G645del-F	5’ -CTTCCCAGAAGATGGAGAG-3’	Genotyping for mutant allele
G645-R	5’ -AGCATGGTAATGAACCTGATATGC-3’	Genotyping
Exon6-F	5’ -GGGAGAACCAACCATGAAGA-3’	qRT-PCR
Exon6-R	5’ -GCTTGCTTCTTGCGATTTTC-3’	qRT-PCR
Exon12-F	5’ -CAAGCCGGAAATTACATGGT-3’	qRT-PCR
Exon12-R	5’ -CTCGCTGCTGAGCCTTTATT-3’	qRT-PCR
18S-F	5’ -GGAAGGGCACCACCAGGAGT-3’	qRT-PCR
18S-R	5’ -TGCAGCCCCGGACATCTAAG-3’	qRT-PCR

Table S1. Primers used for genotyping and qRT PCR.

Antibody	Source, Cat. No	Antibody	Source, Cat. No
RyR2	ENZO, ALX-804-016-R100	NEXN	Custom antibody
JPH2	Santa Cruz, sc-51313	Casq1	Santa Cruz, sc-28274
Casq2	Santa Cruz, sc-390999	SERCA2	Santa Cruz, sc-73022
LTCC	Alomone Labs, ACC-003	GAPDH	Santa Cruz, sc-32233

Table S2. Antibodies used for western blot.
